# Regulation of bone immunity by metal ions: a review of mechanisms and application progress

**DOI:** 10.3389/fimmu.2026.1889301

**Published:** 2026-08-05

**Authors:** Yansong Dong, Zhenqian Qi, Hantao Yang, Xuandu Chen, Yafang Huang, Lei Qin, Jie Tan, Weihong Yi

**Affiliations:** 1 Department of Orthopedic Surgery, Affiliated Nanshan Hospital of Shenzhen University, Shenzhen, China; 2 Department of Orthopedic Surgery, Jiading District Central Hospital, Shanghai, China; 3 Department of Spine Surgery, Southern University of Science and Technology Hospital, Shenzhen, China; 4 Science and Education Department, Wuhan Fourth Hospital, Wuhan, China; 5 Hubei Provincial Sports Medicine Center, Hubei Provincial Clinical Research Center for Orthopaedics, Hubei Key Laboratory of Sports Injury and Precision Therapy, Wuhan, China; 6 Orthopaedic Laboratory, Wuhan Fourth Hospital, Wuhan, China

**Keywords:** biomaterials, bone regeneration, dendritic cells, immunomodulation, macrophage polarization, metal ions, neutrophils, osteoimmunology

## Abstract

The formation and repair of bone tissue depend not only on the activity of bone tissue cells but also on complex regulatory mechanisms mediated by the immune system. With the advancements of osteoimmunology in recent years, the functional roles of metal ions in this field have attracted extensive attention. Various metal ions, including zinc, magnesium, manganese, strontium, and copper, are demonstrated to influence the osteoimmune microenvironment and promote bone repair by modulating the biological functions of immune cells, especially the inflammatory response. However, the precise mechanisms through which metal ions orchestrate osteoimmunity have not been fully elucidated, and the underlying signaling pathways, as well as their internal interactions with cells involved in bone repair, require further in-depth investigation. This review summarizes the regulatory effects of various metal ions on macrophages, T cells, dendritic cells, and neutrophils, including phenotype polarization, maturation, differentiation, inflammatory cytokine expression, and underlying signaling pathways. Besides, this review further discusses the application of metal ion-doped biomaterials in bone tissue engineering, while also covers their functions in remodeling the osteoimmune microenvironment and highlights potential directions for future research directions.

## 1 Introduction

The formation and repair of bone tissue are highly complex biological processes, which involve multiple synergistic interactions with multiple cell types, including osteoblasts, osteoclasts, endothelial cells, immune cells, as well as their surrounding microenvironment. With the fast advancement of osteoimmunology in recent years, researchers have discovered that the immune system plays a central role in bone regeneration, where the changes of immune responses can critically regulate the bone repair process. Various immune cells, including macrophages, dendritic cells, and T cells, are involved in the complex network of the osteoimmune microenvironment. In this immune microenvironment, cytokines and signaling pathways jointly determine the rate and outcome of bone healing, repair, and regeneration (1). Therefore, modulation of the osteoimmune microenvironment has become a research hotspot in the fields of bone tissue engineering.

As reported, metal ions are key regulators of bone metabolism and are widely used in modifying bone substitute materials to accelerate bone regeneration. Various metal ions, such as zinc, magnesium, manganese, strontium, and copper, can directly or indirectly promote the proliferation and differentiation of bone tissue cells and bone matrix deposition through distinct mechanisms. They can also regulate immune cell functions, optimize the osteoimmune microenvironment, and facilitate bone repair (2–4). For example, zinc ions possess favorable antibacterial and antioxidant properties and can induce macrophage polarization toward the pro-healing M2 phenotype via the PI3K/Akt/mTOR signaling pathway, thereby regulating the immune microenvironment and promoting bone formation. Magnesium ions have been shown to promote osteogenesis while inhibiting osteoclast activity, contributing to bone repair by modulating immune responses and angiogenesis (4). Strontium ions, due to their dual effects of promoting bone formation and inhibiting bone resorption, have become a research focus in bone tissue engineering (5, 6). Copper ions exhibit antibacterial activity, regulate immune responses, and promote bone regeneration (7).

In recent years, metal ion-doped biomaterials and functional composite materials have become research hotspots and have demonstrated significant application prospects in bone tissue engineering. Novel materials such as multi-metal organic frameworks and metal-phenolic networks have been rationally designed to achieve controlled release of metal ions and synergistic multifunctional effects, thereby promoting osteoimmunomodulation and bone regeneration (8, 9). For instance, biomimetic periosteal materials containing magnesium and zinc can guide macrophage polarization toward the anti-inflammatory M2 phenotype through sustained release of metal ions, thereby improving the osteoimmune microenvironment and promoting osteogenesis and angiogenesis (10). Magnesium-copper bimetallic organic frameworks integrated with 3D-printed bone scaffolds regulate macrophage polarization and promote angiogenesis and neurogenesis through metal ion release (8). Biomimetic titanium alloy coatings containing zinc and copper ions achieve combined antibacterial, immunomodulatory, and osteogenic effects via staged metal ion release (11, 12). Metal ions can also act synergistically with bioactive molecules to construct composite hydrogels with antioxidant, anti-inflammatory, and immunomodulatory functions, thereby enhancing bone repair outcomes (13, 14). Furthermore, metal ions regulate the production and function of exosomes, influencing immune responses, angiogenesis, and bone formation (15). By virtue of their multiple regulatory functions, metal ions promote bone formation and provide new insights and strategies for the development of bone tissue engineering and clinical bone repair. Future research should aim to clarify the specific targets and signaling pathways of different metal ions, optimize their release patterns and dosages, and integrate them with biomaterials to achieve precise regulation of the osteoimmune microenvironment, thereby advancing the clinical translation and application of bone repair technologies.

## 2 Main body

### 2.1 Basic mechanisms of metal ions in bone immunity

#### 2.1.1 Basic process of bone immunity and participating cells

Bone immunity refers to the dynamic interaction between the immune system and the skeletal system, indicating that bone repair and remodeling are precisely regulated by the immune microenvironment (16, 17). This process is primarily accomplished through the action of immune cells and bone tissue cells via a complex signaling network. When bone injury or infection occurs, neutrophils are the first to be recruited to the injury site. They rapidly clear necrotic tissue and pathogens by releasing reactive oxygen species (ROS) and proteases, initiating the acute inflammatory response (18). Subsequently, a more complex network of immune cells is established. Macrophages play a central regulatory role at this stage, with their functional phenotype polarizing according to the local microenvironment. For example, M1-type macrophages facilitate debridement during the early inflammatory phase, while the subsequent shift to M2-type macrophages suppresses excessive inflammation by secreting anti-inflammatory cytokines (e.g., IL-10, TGF-β) and releases osteogenic growth factors (e.g., BMP-2) and pro-angiogenic factors (e.g., VEGF), directly promoting bone formation and angiogenesis (19–21). Dendritic cells, as antigen-presenting cells, capture antigens at the injury site, migrate to lymph nodes, and activate T cells, thereby bridging innate and adaptive immunity. Once activated, CD4^+^ T cells further regulate macrophage function and bone cell activity through secreted cytokines (22). The balance of different CD4^+^ T helper (Th) cell subsets is particularly critical. For instance, Th1 cells, characterized by IFN-γ secretion, can exert both pro-inflammatory and pro-osteoclastogenic effects (23), whereas Th2 cells, producing IL-4 and IL-13, are generally associated with anti-inflammatory responses and can indirectly promote bone formation (23). Th17 cells, which secrete IL-17A, are potent inducers of osteoclastogenesis and are key drivers of inflammatory bone loss (24), while regulatory T cells (Tregs), through the production of IL-10 and TGF-β, suppress excessive inflammation and inhibit osteoclast formation, thus promoting bone repair (17, 25). Additionally, T follicular helper (Tfh) cells, by supporting B cell responses, indirectly participate in the humoral immune regulation within the bone microenvironment (26). CD8^+^ T cells, traditionally known for their cytotoxic functions, also contribute to bone regulation; they can directly induce bone resorption through the production of pro-inflammatory cytokines like TNF-α and IFN-γ, and some subsets can also express RANKL to promote osteoclastogenesis (22). Furthermore, γδ T cells, which are abundant in mucosal and barrier tissues, act as an early bridge between innate and adaptive immunity. In the context of bone injury or infection, they rapidly respond to pathogens and stress signals, secreting IL-17A and RANKL, thereby significantly contributing to the early inflammatory phase and subsequent osteoclast activation (18, 27).

B cells are not only precursors of antibody-secreting plasma cells but also important regulators of bone metabolism. Through the production of RANKL and OPG, B cells can directly influence osteoclast differentiation and activity. Moreover, they secrete various cytokines (e.g., IL-6, TNF-α, IL-10) that modulate the local immune environment and affect bone homeostasis (28). During bone repair, the infiltration and function of B cells can have dual effects, either promoting or inhibiting osteogenesis depending on their phenotype and the cytokines they produce (29). Natural killer (NK) cells, as key effectors of innate immunity, are also involved in bone immunity. They can recognize and eliminate stressed or infected cells and produce significant amounts of IFN-γ, which not only activates other immune cells but also exerts a potent inhibitory effect on osteoclastogenesis (30), thus potentially limiting bone resorption. Conversely, under certain pathological conditions, NK cells can contribute to bone pathology by promoting inflammation (31).

In the anti-inflammatory, pro-repair microenvironment created by M2 macrophages, Tregs, and Th2 cells, osteoblast activity is enhanced, and new bone matrix is continuously deposited. The RANKL expressed by some T cell subsets (e.g., Th17) and B cells is a key signal for activating osteoclasts and regulating bone resorption, which is crucial for bone homeostasis (32). Concurrently, the immune response gradually subsides, the activity of bone tissue cells returns to basal levels, the bone immune microenvironment restores homeostasis, and bone repair is completed. The intricate interplay among these diverse immune cell subsets determines the ultimate outcome of bone healing and regeneration.

#### 2.1.2 Regulation of immune cell activity and function by metal ions

Metal ions exert broad modulatory effects on diverse immune cells in the bone microenvironment (2). They influence macrophage polarization, T cell subset balance, dendritic cell maturation, and neutrophil effector functions, primarily through regulating intracellular signaling pathways such as NF-κB, MAPK, PI3K/Akt/mTOR, and cGAS-STING (33). Additionally, metal ions interact with metalloregulatory proteins (e.g., metallothioneins, SOD) to modulate oxidative stress and inflammatory responses (34). The following sections detail the specific regulatory mechanisms of each immune cell type, highlighting the distinct roles of different metal ions.

##### 2.1.2.1 Regulation of neutrophils

**Figure 1:**
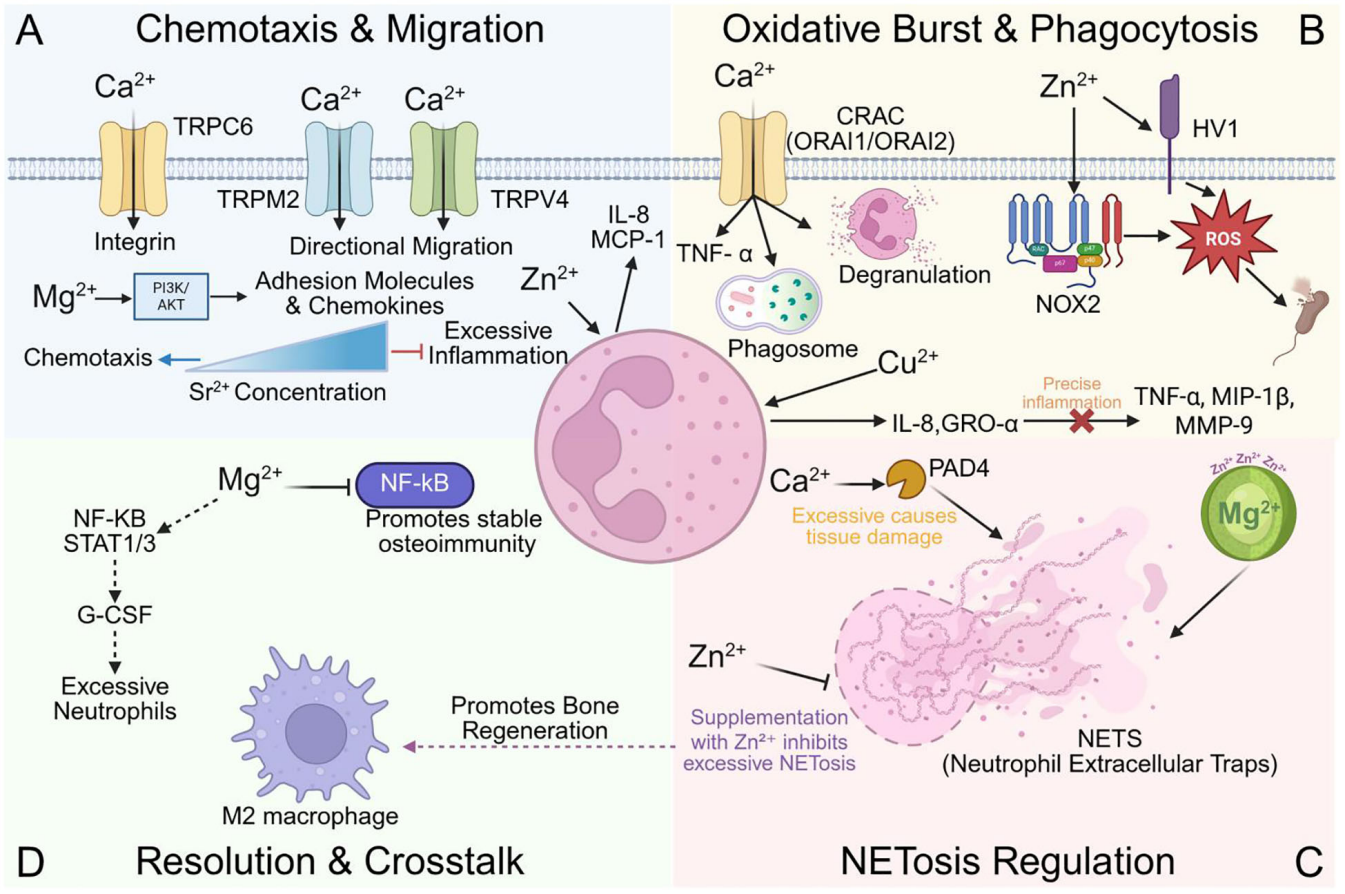
Schematic diagram of the regulatory functions of neutrophils by metal ions **(A)** Chemotaxis & migration; **(B)** Oxidative burst & phagocytosis; **(C)** NETosis Regulation; **(D)** Resolution & crosstalk).

Neutrophils are the most abundant type of white blood cells in circulating human blood. They can be guided by numerous chemokines to migrate to injured or infected bone sites, where they perform immune defense tasks (35), as shown in **Figure 1**.

The TRPC6 channel mediates calcium ion influx during neutrophil chemotaxis, promoting integrin activation and cell adhesion (36). Additionally, the CRAC channel components ORAI1 and ORAI2 regulate calcium signaling in neutrophils, affecting phagocytosis, degranulation, and ROS production, thereby participating in immune defense functions (37). Dynamic changes in calcium ions are also closely related to the production of tumor necrosis factor alpha (TNFα) by neutrophils, with increased calcium enhancing TNFα expression (38). Calcium ions can also regulate the chemotactic ability of neutrophils. Studies have shown that calcium ions promote the migration of neutrophils to inflammatory sites by regulating intracellular signaling pathways (39). Calcium channels such as TRPM2 and TRPV4 play important roles in regulating calcium signaling in neutrophils, affecting their oxidative burst and migration functions (40, 41). Abnormal calcium signaling, such as calcium overload, can lead to excessive production of neutrophil extracellular traps (NETs) by neutrophils, thereby exacerbating tissue inflammation and damage (42, 43). The regulation of neutrophil function by calcium ions in the bone immune environment also involves the coupling of calcium signaling with immunomodulation. For example, calcium-dependent proteases like PAD4 play a crucial role in NET formation, and activation of calcium channels is a prerequisite for inducing PAD4 activity (44). Furthermore, calcium ions can influence the degranulation activity and oxidative stress response of neutrophils, regulating their antibacterial and pro-inflammatory functions (45).

In the bone immune environment, Zn^2+^ has complex effects on the function of NADPH oxidase (NOX2) and the voltage-gated proton channel (HV1). Appropriate release of Zn^2+^ helps maintain ROS production and sustain the oxidative burst, thereby enhancing the bactericidal ability of neutrophils (46). For example, Zn^2+^-containing biomaterials can continuously release zinc ions, promoting ROS generation in neutrophils and enhancing their efficiency in clearing bacteria, thus effectively preventing implant-related infections (47). Furthermore, the regulation of NETosis by Zn^2+^ is also critical. In mouse bone defect repair, overactive NETosis leads to persistent inflammation and delayed bone healing. Studies have successfully suppressed excessive NETosis by supplementing Zn^2+^, promoting M2 macrophage polarization and significantly improving bone regeneration outcomes (48). This indicates that Zn^2+^ not only enhances the direct antibacterial function of neutrophils but also indirectly regulates the inflammatory response of immune cells. Zinc ions control the chemotactic migration of neutrophils by regulating the release and expression of chemokines such as IL-8 and MCP-1. Research has found that abnormal intracellular zinc ion concentration and transporter expression can lead to reduced IL-8 secretion, thereby affecting immunomodulation and inflammatory response capacity (49). This suggests that zinc ions participate in regulating neutrophil function in the bone immune environment by modulating chemokine expression.

Copper ions can regulate the function of neutrophils (PMNs). Studies have found that copper ion-doped biomaterials, such as copper-doped biphasic calcium phosphate (BCP), can modulate the release of inflammatory factors from neutrophils, thereby regulating the immune microenvironment. Copper-doped BCP materials increased the secretion of IL-8 and GRO-α *in vitro*, but did not significantly affect the secretion of other inflammatory factors like MIP-1β, TNF-α, and MMP-9. This indicates that copper ions can precisely regulate the release of inflammatory factors from neutrophils, inducing a moderate inflammatory response that is beneficial for the recruitment and activation of immune cells and accelerating bacterial clearance at the infection site (50).

In the bone immune microenvironment, Mg^2+^ promotes bone tissue repair and regeneration by regulating neutrophils. First, magnesium ions help maintain normal immune homeostasis. Studies show that Mg^2+^ deficiency causes a rise in systemic inflammatory levels, manifested by increased blood neutrophils and activation of inflammatory signaling pathways such as NF-κB, STAT1, and STAT3, suggesting that Mg^2+^ may participate in immunomodulation through these signaling pathways (51). In addition, magnesium ions significantly affect the chemotaxis and function of neutrophils. Controlled dietary magnesium intake can induce an increase in granulocyte progenitors in the bone marrow and peripheral blood neutrophilia, accompanied by enhanced G-CSF production and activation of the STAT3 signaling pathway, thereby promoting neutrophil differentiation and proliferation (52). In signal transduction, Mg^2+^ regulates the PI3K/Akt pathway. Activation of the PI3K/Akt pathway can regulate the expression of intracellular adhesion molecules and chemokines, further enhancing the responsiveness of neutrophils to inflammatory signals. Moreover, Mg^2+^-doped materials can also enhance the antibacterial ability of neutrophils. For example, a zinc-doped ferric oxyhydroxide nanolayer on the surface of magnesium alloys can promote the formation of NETs, enhancing antibacterial activity and promoting osseointegration (53). Neutrophils capture and kill bacteria by releasing NETs, a process closely related to the regulation of magnesium ions (54). Additionally, magnesium ions can regulate the chemotactic ability and oxidative burst function of neutrophils, affecting their phagocytic and bactericidal efficacy (55); magnesium ions can also inhibit the NF-κB signaling pathway, reducing inflammatory responses and promoting the stability of the bone immune environment, thereby facilitating effective bone tissue repair (56, 57).

The concentration gradient of strontium ions also significantly affects neutrophil chemotaxis. Low concentrations of strontium ions exert immunomodulatory effects, enhancing immune regulation during bone healing and promoting the timely migration of neutrophils into bone defect sites to participate in initial immune responses and tissue repair processes (5). Conversely, excessively high strontium ions can lead to dysfunction of immune cells, suppressing excessive inflammatory responses and avoiding tissue damage caused by the aggregation of large numbers of neutrophils.

In summary, Ca^2+^, Zn^2+^, Cu^2+^, Mg^2+^, and Sr^2+^ all modulate neutrophil functions—including chemotaxis, NETosis, and oxidative burst—but through distinct and sometimes opposing mechanisms. Ca^2+^ and Mg^2+^ generally promote chemotaxis and ROS production, whereas Zn^2+^ uniquely suppresses excessive NETosis while enhancing bactericidal oxidative burst; Cu^2+^ fine-tunes the release of specific inflammatory cytokines, and Sr^2+^ exerts concentration-dependent effects, with low levels promoting chemotaxis and high levels dampening excessive inflammation. These differences highlight that the immunomodulatory outcome of metal ions is highly dependent on their identity, concentration, and downstream signaling pathways (e.g., calcium signaling, PI3K/Akt, NF-κB, and STAT3).

##### 2.1.2.2 Regulation of macrophages

**Figure 2:**
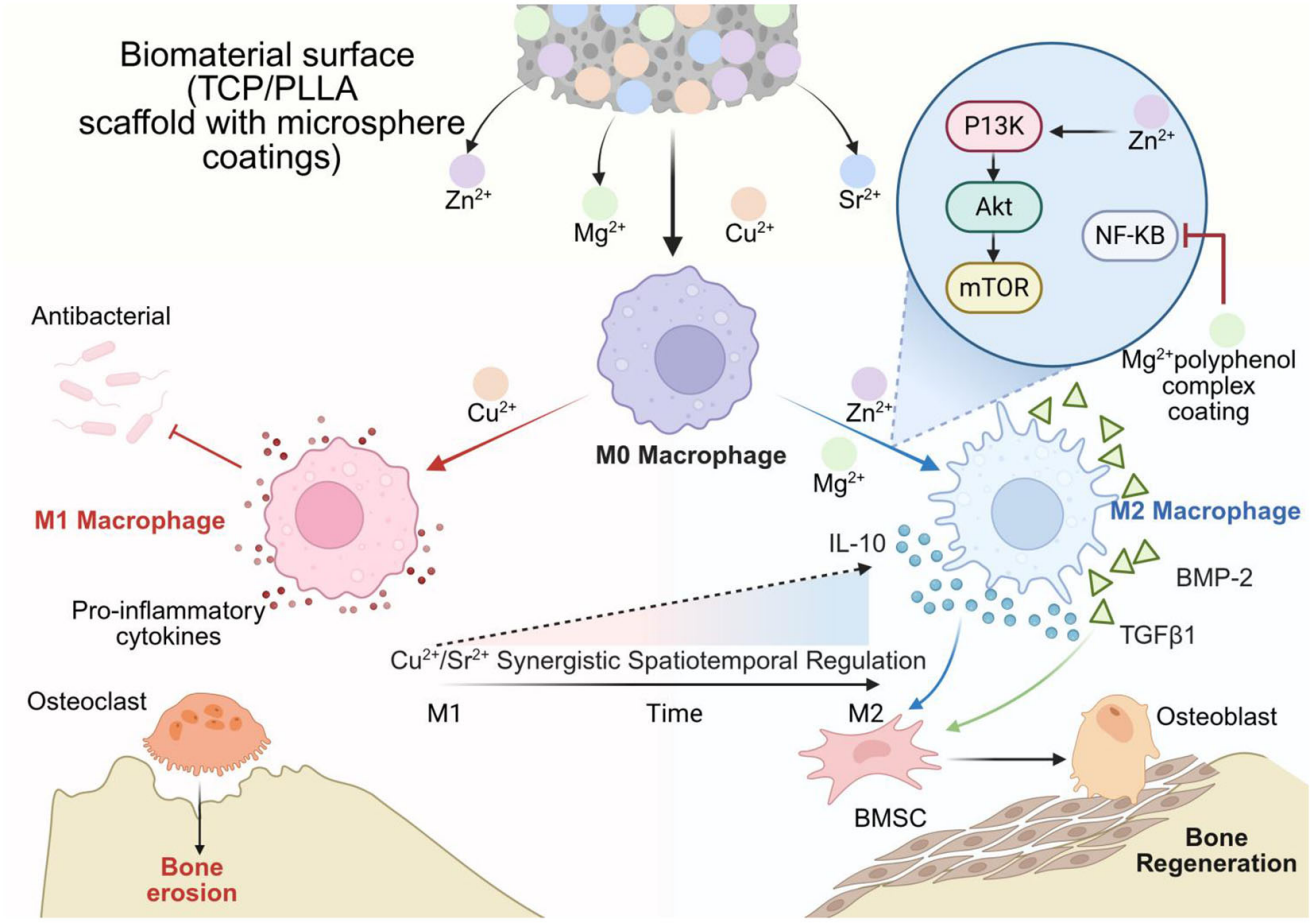
Schematic of metal ion-mediated macrophage polarization, regulating the balance between M1 (pro-inflammatory, bone-eroding) and M2 (anti-inflammatory, bone-repairing) phenotype.

Macrophages are mainly divided into two functional phenotypes: M1-type and M2-type. M1-type macrophages promote inflammatory responses, aiding in the clearance of lesions and damaged tissue. However, overactivation of M1 macrophages enhances osteoclast activity while simultaneously inhibits osteoblasts, leading to bone turnover imbalance. In contrast, M2-type macrophages promote the resolution of inflammation, thus accelerating bone tissue repair (58). The schematic diagram of the biomaterial surface modification and its immunomodulatory effects on macrophage polarization is shown in **Figure 2**. In recent years, metal ions such as zinc (Zn^2+^) and magnesium (Mg^2+^) have been shown to induce macrophage polarization towards the M2 phenotype. For example, a controlled release system for magnesium ions based on PLGA/MgO-alendronate microspheres can induce macrophage polarization from a non-polarized state (M0) to the M2 type, increasing the secretion of anti-inflammatory cytokines (IL-10) and osteogenic factors (BMP-2, TGF-β1), thereby promoting bone regeneration (56). Zinc ion-releasing beta-tricalcium phosphate/polylactic acid (TCP/PLLA) composite scaffolds have been shown to promote macrophage polarization to M2 by activating the PI3K/Akt/mTOR signaling pathway (3). Composite coatings formed by magnesium and zinc ions with polyphenols can synergistically inhibit macrophage polarization towards M1 and promote their shift to M2, reducing inflammation and enhancing the osteogenic differentiation of bone marrow mesenchymal stem cells (59). Additionally, copper ions can promote M1 polarization to initiate antibacterial immune responses but simultaneously synergize with strontium ions to promote the secretion of osteogenic factors, achieving immunomodulatory osteogenesis (60). Iron ions can also regulate macrophage polarization; iron overload typically promotes M1 polarization and increases ROS production (61), while iron restriction can induce M2 polarization, supporting tissue repair (62). This indicates that the regulation of different metal ions, their release concentrations, and timing can achieve dynamic control of macrophage polarization to meet the different stages of bone tissue healing.

##### 2.1.2.3 Regulation of T cells

**Figure 3:**
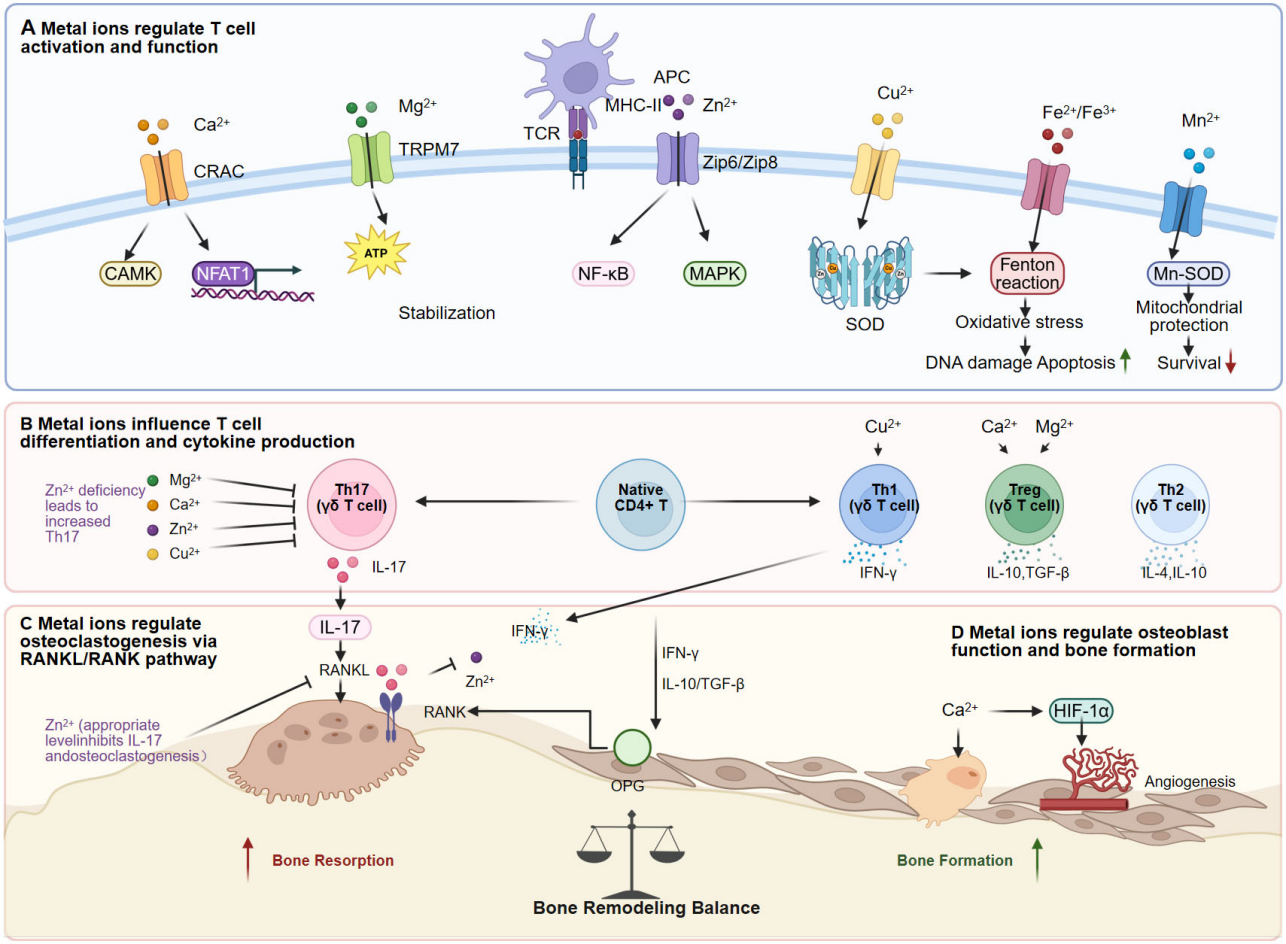
Schematic illustration of metal ions regulating T cell function. **(A)** Metal ions regulate T cell activation and function; **(B)** Metal ions influence T cell differentiation and cytokine production; **(C)** Metal ions regulate osteoclastogenesis via RANKL/RANK pathway; **(D)** Metal ions regulate osteoblast function and bone formation).

T cells are mainly divided into CD4^+^ T cells, CD8^+^ T cells, and γδ T cells. Different T cell subsets have distinct functions. Different metal ions regulate the bone immune microenvironment and promote bone regeneration and repair by influencing T cell subset ratios and cytokine secretion (17, 32, 63). The schematic diagram illustrating how metal ions regulate T cell signaling, subset differentiation, and subsequent effects on osteoclasts and osteoblasts is shown in **Figure 3**.

Regarding the regulation of classical T cell subsets, when T cell receptors (TCR) recognize antigens and become activated, calcium ions act as intracellular second messengers, controlling T cell proliferation, differentiation, and cytokine secretion, thus determining the effector functions of T cells (64, 65). Studies have found that serum calcium levels are positively correlated with CD4^+^ T cells and Tregs but negatively correlated with CD8^+^ T cells (66). Calcium ions can also regulate the balance between Th17 cells and Tregs. IL-17A secreted by Th17 cells promotes osteoclastogenesis, while calcium ions can enhance the immunomodulatory function of Tregs, thereby increasing osteoblast activity. For example, biphasic calcium phosphate materials recruit CD4^+^ T cell populations, primarily Tregs, by activating calcium channels in macrophages, which is beneficial for bone formation (67). Simultaneously, calcium ions can induce the production of the angiogenic factor HIF-1α, improving blood circulation around bone defects (68).

Zinc ions influence T cell function by modulating TCR signaling intensity. TCR activation increases the concentration of zinc ions in the cytoplasm, and silencing Zip6 inhibits zinc ion influx and T cell responses. Zinc ions regulate T cell immune responses through signaling pathways such as NF-κB and MAPK (69). Studies have shown that zinc deficiency impairs the function of Tregs while increasing the number of pro-inflammatory Th17 cells, leading to an imbalance in bone immunity (70).

Magnesium ions (Mg^2+^) affect T cell activation and proliferation by regulating the transient receptor potential melastatin 7 (TRPM7) channel (71). Magnesium ions can enhance the glycolytic and oxidative phosphorylation metabolism of CD8^+^ T cells, restoring their metabolic activity (72). Magnesium ions can enhance the immunosuppressive function of Tregs and inhibit the activity of pro-inflammatory cells. For example, magnesium ions released from magnesium-aluminum layered double hydroxides not only promote the transformation of macrophages to the anti-inflammatory M2 phenotype but also effectively promote the recruitment of Tregs while inhibiting the activity of pro-inflammatory Th17 cells and plasma cells (73).

Copper ions, as cofactors for SOD, can protect T cells from oxidative damage. Copper deficiency leads to impaired function of helper T cells (Th) and cytotoxic T lymphocytes (CTLs) (74). Studies have found that copper ions can promote the expression of T cell-related cytokines such as IL-2 (75). Copper ions exert important effects on osteoclast activity and bone resorption by regulating T cell-mediated inflammatory responses. Research has shown that copper-doped bioactive glasses can significantly modulate the immune phenotype and cytokine secretion of T cells, promoting an increase in the proportion of Th1 cells while inhibiting the number of Th17 cells (76).

In addition to the aforementioned classical T cell subsets, γδ T cells, as important components of the bone immune microenvironment, are also finely regulated by various metal ions. Calcium ions, also acting as second messengers, promote γδ T cell proliferation and secretion of cytokines such as IFN-γ and IL-17 via the CRAC-NFAT pathway, and may influence osteoclast differentiation through the RANKL/RANK pathway, thereby participating in osteoporosis and inflammatory bone diseases (77, 78). Zinc ions serve as cofactors for various enzymes and transcription factors (e.g., NF-κB); their deficiency leads to impaired maturation and reduced cytotoxic function of γδ T cells, while appropriate zinc levels can inhibit excessive IL-17 production by γδ T cells, reduce osteoclastogenesis, and exert anti-inflammatory and bone-protective effects (79–81). Magnesium ions, as cofactors for integrins (e.g., LFA-1), affect the adhesion and migration of γδ T cells to bone tissue; hypomagnesemia may promote their differentiation toward a pro-inflammatory Th17 phenotype, exacerbating bone resorption (82, 83). Copper ions participate in the composition of SOD to regulate the antioxidant capacity of γδ T cells, but excess copper may inhibit their proliferation (84, 85). In addition, iron ions (Fe^3+^/Fe^2+^) have a dual role: they are essential for cellular respiration, but excess iron can induce oxidative stress via the Fenton reaction, damaging γδ T cell membranes and DNA; under iron overload conditions, γδ T cell apoptosis increases and osteoclast activity is enhanced, promoting osteoporosis (86). Manganese ions (Mn^2+^), as cofactors for Mn-SOD, protect γδ T cells from mitochondrial oxidative damage, favoring their survival (87). In summary, different metal ions form a complex regulatory network in the bone immune microenvironment through multidimensional modulation of various T cell subsets (including CD4^+^, CD8^+^, and γδ T cells). A deeper understanding of these mechanisms will provide important theoretical foundations for metal ion-based strategies in bone regeneration and the treatment of bone diseases (22, 88).

##### 2.1.2.4 Regulation of dendritic cells

**Figure 4:**
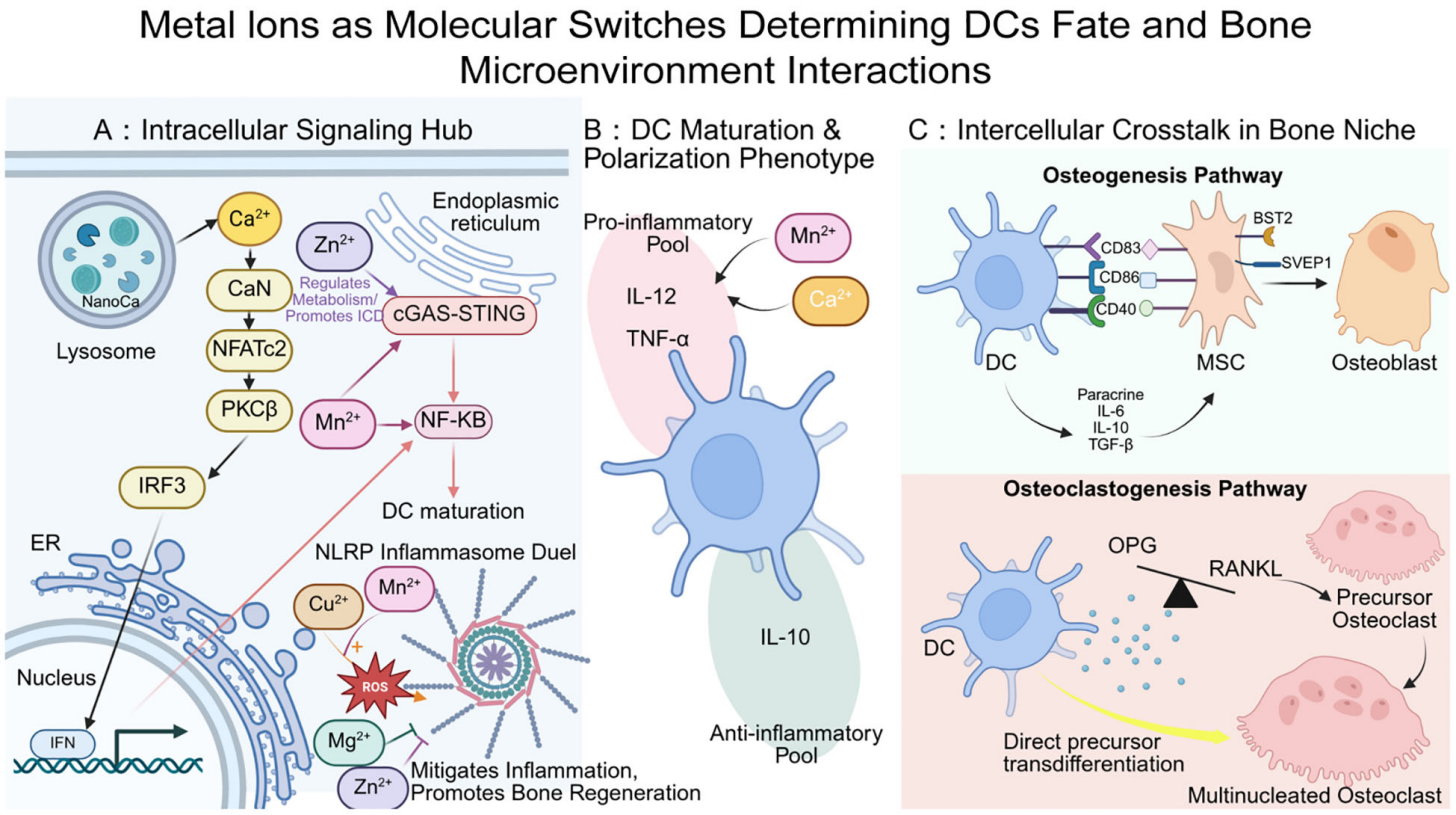
Schematic illustration of metal ions regulating DCs. **(A)** Intracellular signaling hub; **(B)** DC maturation & polarization phenotype; **(C)** Intercellular crosstalk in bone niche).

Dendritic cells (DCs) are crucial immune regulators. Their activation state and secretion of various cytokines can regulate the balance between bone formation and resorption. The schematic diagram illustrating how metal ions (Ca^2+^, Zn^2+^, Mg^2+^, Mn^2+^, Cu^2+^) regulate DC maturation, polarization, inflammasome activation, and subsequent effects on the bone microenvironment is shown in **Figure 4**.

DCs can influence mesenchymal stem cells (MSCs) by secreting pro-inflammatory or anti-inflammatory cytokines. For example, cytokines secreted by DCs, such as IL-6, IL-10, and TGF-β, can regulate MSC function and their differentiation potential into osteoblasts (89). Research has shown that molecules on the surface of DCs, such as CD83, CD86, and CD40, not only affect the secretory function of MSCs but also directly impact the osteogenic differentiation ability of MSCs (90).

Studies indicate that certain metal ions can influence the effect of DCs on osteoclastogenesis by affecting their maturation, secretory function, and immune activity. For instance, metal ions may regulate the expression of RANKL and OPG in DCs, altering their ability to induce osteoclast differentiation (91). DCs can also act as precursor cells for osteoclasts, participating in bone metabolism. Research has found that certain DC subsets can both differentiate into mature DCs and transform into osteoclasts (92, 93).

Metal ions can promote the maturation and activation of DCs through different mechanisms. For example, Ca^2+^, as a second messenger, can promote the M1-type macrophage polarization of DCs and their maturation (94). One study found that a calcium-containing phospholipid nanomaterial (NanoCa) can accelerate Ca^2+^ release via the lysosomal pathway and activate the Ca^2+^-calcineurin (CaN)-NFATc2-PKCβ-IRF3 signaling pathway, promoting interferon production and enhancing DC maturation and antiviral/anti-tumor capabilities. Mn^2+^ promotes DC maturation by activating the STING pathway (95, 96). Mn^2+^ can also be used to synthesize nanocomplexes that promote the maturation and antigen cross-presentation of BMDCs via the NF-κB pathway (97). The release of Zn^2+^ can regulate metabolic pathways, promote immunogenic cell death (ICD), and thereby enhance DC maturation and function (98); Zn^2+^ can also regulate the secretion of cytokines such as TNF-α and IL-6 by DCs (99). Zn^2+^ can also activate the cGAS-STING signaling pathway, promoting DC maturation (100, 101).

Metal ions can regulate the balance between pro-inflammatory and anti-inflammatory cytokines in the immune microenvironment by modulating the cytokine secretion of DCs. Metal ions such as Mn^2+^ and Ca^2+^ can promote DCs to secrete pro-inflammatory cytokines like IL-12 and TNF-α, and can also affect the expression of anti-inflammatory factors like IL-10. For example, a manganese-based immunostimulatory metal-organic framework (ISAMn-MOF) can significantly activate the cGAS-STING signaling pathway in DCs, enhancing their ability to secrete pro-inflammatory cytokines (102). Metal ions can activate the NLRP3 inflammasome by affecting intracellular ROS levels and lysosomal stability. For instance, copper-manganese silicate nanoparticles activate the NLRP3 inflammasome in DCs under high ROS environments, promoting their maturation and antigen presentation, thereby enhancing immune responses (103). Furthermore, Mg^2+^ and Zn^2+^ alleviate inflammatory responses and promote bone regeneration by interfering with NLRP3-related pathways (73, 104). This suggests that metal ions regulate DC-mediated immune-inflammatory responses and subsequently affect bone metabolism by modulating the NLRP3 inflammasome.

#### 2.1.3 Influence of metal ions on inflammatory cytokines and immune signaling pathways

Metal ions regulate immune cell functions via NF-κB, MAPK, STAT, and mTOR pathways, controlling inflammation, polarization, survival, and death. In NF-κB, Ti^4+^ activates it in macrophages, inducing TNF-α and inflammation via Hippo-YAP crosstalk (105); Ti also exacerbates inflammation through this crosstalk (105). Mn^2+^ promotes DC and TAM maturation via NF-κB and cGAS-STING, enhancing anti-tumor responses (106, 107). In MAPK, p38 mediates CTSC-driven M1 polarization via FAK, promoting inflammatory factors (108). STAT is involved; Mg/Zn membranes induce M2 via JAK-STAT for bone regeneration (10), while copper bidirectionally regulates macrophage activity, likely through multiple STAT pathways (109). In mTOR, Ce/Zn composites restore mitochondrial metabolism, balance M1/M2, reduce NF-κB p65 activity, and promote bone formation (15). Metal-induced ROS cause oxidative stress affecting apoptosis/pyroptosis; Mn nanomaterials activate cGAS-STING and NF-κB to induce immunogenic cell death (106), and copper modulates oxidative stress to balance pathogen clearance and potential escape (109). Apoptosis releases inflammatory mediators, thereby regulating the bone immune microenvironment.

### 2.2 Specific roles of different metal ions in bone immunomodulation

#### 2.2.1 Zinc ions

Zinc ions (Zn^2+^), as an essential trace element for the human body, play numerous key roles in bone immunomodulation and bone regeneration processes. Zinc ions promote the differentiation of bone marrow MSCs into osteoblasts. Relevant studies indicate that incorporating zinc ion sustained-release systems into bone repair materials enhances the osteogenic differentiation ability of MSCs. For instance, a multifunctional zinc ion sustained-release coating composed of carboxylated graphene oxide, zinc ions, and a chitosan layer can regulate zinc ion release according to different stages of bone fusion: promoting immunomodulation during early release, angiogenesis during the middle stage, and osseointegration during the late stage, thereby improving trabecular bone thickness and mechanical interlocking strength (110). Additionally, zinc ions promote macrophage polarization towards the M2 phenotype by activating the PI3K/Akt/mTOR signaling pathway, alleviating local inflammatory responses and benefiting bone repair (3, 48). Both *in vitro* and animal model studies have confirmed that zinc ions promote M2 macrophage polarization, reduce inflammation, and enhance bone regeneration.

Zinc ions also synergistically promote angiogenesis to support the bone regeneration process. Zinc ions can induce angiogenesis in human umbilical vein endothelial cells (HUVECs) and inhibit osteoclastogenesis. By promoting the osteogenic effect of mBMSCs and the angiogenesis of HUVECs, zinc ions create a favorable environment for improving bone defect repair and bone regeneration. Various zinc-containing bone repair materials achieve effective bone defect repair by regulating immune responses, promoting angiogenesis, and facilitating bone formation (111, 112). For example, zinc-containing hydroxyapatite composites promote the osteogenic differentiation of MSCs while also creating a pro-osteogenic immune environment by regulating the immune phenotype of macrophages (113). Furthermore, sustained-release systems for zinc ions can reduce local oxidative stress and inflammatory responses, providing a favorable microenvironment for bone healing. In summary, zinc ions effectively regulate bone immunity and promote bone regeneration through the synergistic effects of promoting MSC differentiation into osteoblasts, inducing macrophage polarization towards the M2 phenotype to reduce inflammation, and promoting angiogenesis.

#### 2.2.2 Magnesium ions

Magnesium ions (Mg^2+^) are crucial components of bone homeostasis and metabolism. They regulate macrophage function through various mechanisms to maintain bone immune homeostasis, inhibit inflammatory responses, and promote osteogenesis and osseointegration.

Magnesium ions can regulate macrophage polarization to maintain bone immune homeostasis. Research has shown that Mg^2+^-controlled tissue microenvironments can effectively guide macrophage polarization from M0 to the M2 phenotype by increasing the production of anti-inflammatory cytokines (IL-10) and osteogenic factors (BMP-2 and TGF-β1), creating a bone immune microenvironment favorable for the proliferation and osteogenic differentiation of bone marrow mesenchymal stem cells (82, 114). During the early inflammatory phase, Mg^2+^ upregulates the expression of transient receptor potential cation channel member 7 (TRPM7), ultimately leading to the phosphorylation of histone H3 at inflammatory cytokine promoters, forming a pro-osteogenic immune microenvironment (82). This regulatory mechanism highlights the crucial role of magnesium ions in promoting the restoration of bone immune homeostasis by modulating macrophage signal transduction during the early stages of bone repair.

Magnesium ions can inhibit the NF-κB signaling pathway, reducing early inflammatory responses and thereby promoting osteogenesis. NF-κB, a key regulator of inflammatory and immune responses, can lead to chronic inflammation and inhibit osteogenesis when overactivated. Magnesium ions upregulate integrin α5β1 and inhibit Toll-like receptors, thereby mediating macrophage immune responses, suppressing the NF-κB signaling pathway, reducing the expression of inflammatory cytokines such as TNF-α and IL-6, while promoting the production of anti-inflammatory cytokines like IL-10 and TGF-β1. This induces macrophage polarization towards the M2 phenotype, establishing an immune microenvironment conducive to bone regeneration (56, 114, 115). For example, magnesium-containing nanomaterials successfully inhibited inflammatory responses and promoted the osteogenic differentiation of bone marrow mesenchymal stem cells by reducing NF-κB expression activity (56). In bone defect models, magnesium-based composites accelerated bone healing by inhibiting NF-κB signaling (115). This immunomodulatory effect provides a molecular basis for magnesium ions promoting osteogenesis.

Magnesium and its alloys, as a class of degradable and absorbable biomaterials, offer unique advantages for promoting osseointegration. Magnesium-based alloys and their composites possess excellent biocompatibility, mechanical properties, and controllable degradation rates, making them a research hotspot in the field of bone defect repair. The Mg^2+^ released during degradation directly participates in bone metabolism and also regulates the local immune microenvironment, promoting new bone formation and angiogenesis (116–118). For instance, magnesium-based artificial dressings promote cortical bone formation by releasing Mg^2+^, activating M2 polarization of periosteal macrophages, promoting angiogenesis and nerve growth, thereby achieving rapid repair of bone defects (119). Additionally, 3D-printed magnesium-doped bioactive glass scaffolds exhibit excellent bone regeneration and immunomodulatory functions (117). Strategies such as mineralization with collagen address the issue of rapid degradation of magnesium alloys, improving their corrosion behavior and bioactivity, and enhancing bone repair outcomes (120). These findings indicate that magnesium-based degradable implants provide mechanical support and also regulate local immunity through Mg^2+^ release, promoting osseointegration.

#### 2.2.3 Manganese ions

Manganese (Mn^2+^), a transition metal exhibiting multiple valence states and high-spin characteristics, is a key cofactor for osteogenic enzymes such as alkaline phosphatase. It participates in redox regulation, antioxidant defense, osteogenesis, and angiogenesis through its biological functions. Manganese possesses various catalytic functions, activating multiple oxidases to scavenge excess ROS and regulate immune-inflammatory responses, ultimately promoting bone regeneration. Notably, it exhibits a prominent immune-enhancing effect in promoting dendritic cell maturation and antigen cross-presentation. Dendritic cells bridge innate and adaptive immunity, and their maturation status directly impacts effective antigen presentation and T cell activation. Studies have shown that manganese-based nanomaterials can promote the maturation of BMDCs, enhance their antigen uptake capacity and secretion of pro-inflammatory cytokines, and improve the efficiency of antigen cross-presentation. This is crucial for activating specific T cell immune responses and plays a key role in anti-tumor and anti-infection immunity (121, 122). Mechanistically, manganese ions can activate multiple immune signaling pathways, including the cGAS-STING pathway, a key pathway for innate immune sensing of DNA. Manganese ions enhance the activity of the cGAS enzyme, promoting the production of cyclic GMP-AMP, activating STING, and inducing the expression of type I interferons and various other pro-inflammatory cytokines. This signal activation promotes dendritic cell maturation and enhances adaptive immune effects, such as activating cytotoxic T lymphocytes and regulating helper T cell polarization, forming a more effective immune response (97, 123). Furthermore, manganese-based metal-organic frameworks, as novel immunostimulatory materials, can continuously release Mn^2+^, promoting immune cell infiltration and functional enhancement by activating multiple signaling pathways, demonstrating excellent efficacy in cancer immunotherapy (123).

In the field of bone tissue repair, Mn^2+^ can regulate adaptive immune response processes, modulate the phenotypic shift of T lymphocyte polarization, and promote the osteogenic differentiation of BMSCs. Relevant studies have demonstrated that manganese-doped silicate-hydroxyapatite nanowires can regulate the balance of CD4^+^ T cell subsets, significantly inducing a shift towards helper Th2 polarization while inhibiting the maturation of helper Th1 cells, ultimately establishing an immunomodulatory microenvironment favorable for bone tissue formation. This regulation of the Th1/Th2 balance is mainly achieved through the activation of manganese superoxide dismutase (MnSOD) and AMP kinase (AMPK) signaling pathways, amplifying the osteogenic regulatory effect through downstream cascading reactions and enhancing the efficiency of bone defect repair (124). In addition to modulating CD4^+^ T helper cell balance, manganese ions also influence CD8^+^ T cell responses. Mn^2+^ has been shown to enhance the activation and effector function of CD8^+^ cytotoxic T lymphocytes (CTLs) through the cGAS-STING pathway, increasing their production of granzyme B and IFN-γ (125–127). This effect is critical for anti-tumor and anti-infection immunity within the bone microenvironment. Furthermore, Mn^2+^ promotes the metabolic fitness of CD8^+^ T cells by supporting mitochondrial respiration, thereby sustaining their long-term survival and cytotoxic capacity (128, 129). Additionally, manganese ions can regulate macrophage polarization towards the anti-inflammatory M2 type, effectively reducing the intensity of local inflammatory responses and optimizing the immune environment for bone regeneration (130, 131).

#### 2.2.4 Strontium ions

Strontium (Sr^2+^) is a trace element present in the human body. Research indicates that strontium plays a crucial role in the homeostasis of osteoclasts and osteoblasts, regulating the balance of bone metabolism. Strontium ions can regulate immune responses, exerting positive effects on macrophage function and triggering a favorable bone immune microenvironment that aids bone healing.

Strontium ions can regulate macrophage polarization, promoting the formation of M2-type macrophages. M2-type macrophages exert anti-inflammatory and immunomodulatory effects by secreting various anti-inflammatory cytokines such as IL-4, IL-10, and TGF-β, thereby accelerating the resolution of inflammation and promoting bone repair. Multiple studies have shown that strontium-containing biomaterials or strontium ion release systems can effectively induce macrophage polarization from the M0 to the M2 phenotype, inhibit the secretion of inflammatory cytokines, improve the local inflammatory environment, and enhance the osteogenic differentiation of BMSCs. For example, strontium-doped mesoporous bioactive glass carriers combined with a metformin (MET) release system promote macrophage differentiation towards the M2 type, inhibit excessive ROS generation, reduce oxidative stress, create a favorable immune microenvironment for diabetic bone defects, and improve bone repair outcomes (132). Furthermore, strontium-doped calcium phosphate ceramics (SrTCP) and strontium ion-functionalized collagen membranes also rely on inducing M2 macrophage polarization to optimize the immune microenvironment in bone defect areas and promote bone regeneration (133, 134). In this process, strontium ions regulate macrophage phenotype switching via multiple signaling pathways such as JAK-STAT and MAPK, accelerating bone tissue repair and regeneration.

Strontium ions directly promote the proliferation and differentiation of osteoblasts by activating the calcium-sensing receptor (CaSR) and osteogenesis-related signaling pathways, thereby promoting bone formation. CaSR, a key receptor for sensing extracellular calcium ion concentration, can also sense strontium ions and activate downstream osteogenic signaling pathways. Studies have shown that strontium ions can activate pathways such as Wnt/β-catenin, MAPK, and PI3K/AKT to promote BMSC osteogenesis. For example, functionalized strontium-silk fibroin-hydroxyapatite composite scaffolds, upon Sr^2+^ release, stimulate the expression of pro-healing related cytokines, subsequently activating relevant signaling pathways. This is specifically manifested by promoting macrophage polarization towards M2, promoting angiogenesis and bone regeneration, and enhancing graft-host bone integration (135, 136).

Strontium ions can inhibit bone resorption and participate in maintaining bone metabolic balance. By promoting osteoblast proliferation and differentiation and inhibiting osteoclast activity, strontium ions reduce bone loss and protect bone mass. Research indicates that strontium-doped materials can reduce local inflammatory responses and pro-inflammatory cytokine levels, regulating the bone immune environment to inhibit osteoclast activity (137, 138). This dual mechanism of immunomodulation and direct action on osteoclasts makes strontium ions an ideal element for treating osteoporosis and bone defects.

#### 2.2.5 Copper ions

Copper ions, as one of the many trace elements in the human body, play a crucial role in bone immunomodulation and bone tissue regeneration. Firstly, copper ions promote angiogenesis, enhancing the nutritional supply to bone tissue. Clinically, poor vascularization of bone tissue is a key factor affecting bone formation in patients receiving radiotherapy. Research shows that copper-containing materials can improve bone formation by enhancing angiogenesis. For instance, Ti6Al4V-1.5Cu alloy prepared using selective laser melting technology releases copper ions to promote angiogenesis while inhibiting the release of inflammatory factors, improving vascularization in irradiated bone defect models, partly through the regulation of the PI3K-Akt signaling pathway (139). Additionally, composite materials with synergistic effects of copper ions and silicon ions can promote angiogenesis in bone defects and reduce local inflammatory responses, effectively promoting bone tissue repair (140).

Copper ions participate in immunomodulation and play a key role in macrophage polarization. Copper ions can promote the transformation of macrophages towards the anti-inflammatory M2 phenotype. The anti-inflammatory factors secreted by M2-type macrophages are beneficial for tissue repair and bone regeneration. For example, Ti-5Cu alloy, due to the addition of copper, possesses certain antibacterial functions. Research indicates it has good biocompatibility, promotes macrophage polarization to M2, and accelerates bone regeneration by secreting Oncostatin M (OSM) to activate the osteogenic differentiation signal in osteoblasts (141). Copper-doped bioactive glasses and copper ion-doped biphasic calcium phosphate materials also exhibit the ability to modulate immune responses, such as inducing macrophages to produce bone-promoting factors and regulating the local inflammatory environment, thereby promoting bone repair (50, 142). Copper ions can also regulate T cell subsets; for example, appropriate copper ion concentrations can increase the proportion of Th-1 cells while decreasing the level of Th17 cells, promoting interaction between immune cells and optimizing the immune microenvironment for bone regeneration (76).

Copper ions are widely used on implant surfaces to enhance their antibacterial properties and osseointegration ability. Copper ions possess a “contact killing” antibacterial effect, inhibiting bacterial growth such as Staphylococcus aureus and Escherichia coli. They can also improve the pathological immune microenvironment, enhance local antibacterial activity, and promote local bone regeneration by inducing immune responses in macrophages (60, 143). For example, a copper/procyanidin coating prepared on a titanium alloy surface using dopamine hydrochloride can inhibit the growth of S. aureus and E. coli. The small amount of copper ions released can promote the transformation of macrophages from the pro-inflammatory M1 to the anti-inflammatory M2 type, regulating the secretion of osteogenic factors by bone marrow mesenchymal stem cells and promoting bone formation, demonstrating immunomodulatory and antibacterial properties (144).

#### 2.2.6 Cerium ions

Cerium ions (Ce^3+^/Ce^4+^), a rare earth metal ion, hold great promise for bone immunomodulation and osteogenic effects. Due to the reversible interconversion between the two oxidation states, cerium ions exhibit excellent antioxidant capacity. In cerium oxide (CeO_2_), cerium ions form oxygen vacancies by gaining or losing electrons, displaying activities similar to superoxide dismutase (SOD) and catalase (CAT). These enzyme-like activities enable cerium oxide nanoparticles (CeNPs) to effectively decompose and scavenge free radicals such as ROS and reactive nitrogen species (RNS), reducing cellular oxidative stress levels and maintaining the functional stability of bone immune-related cells and their microenvironment. Experiments have shown that using different ligands to regulate the surface Ce^3+^/Ce^4+^ ratio can effectively tune its enzyme-like activity. For example, the addition of the triethyl phosphonate (TEP) ligand promoted the formation of oxygen vacancies, thereby enhancing SOD-like activity (145). Cerium ion-loaded bioactive glasses, through the sustained release of Ce^3+^/Ce^4+^, demonstrate a good ability to catalytically decompose H_2_O_2_, protecting bone-related cells from oxidative damage and promoting the formation of bone-like structures (146). Doping with transition metals such as copper has also been shown to reduce the temperature required for Ce^4+^ to Ce^3+^ conversion and increase the stability of Ce^3+^, optimizing antioxidant capacity (147).

In the form of cerium oxide nanoparticles (CeO_2_ NPs), cerium ions exhibit strong antibacterial properties, especially for infection control under acidic pathological conditions. Studies have shown that cerium oxide nanoparticles can release active cerium ions (Ce^3+^ and Ce^4+^) under acidic conditions to exert bactericidal effects. For example, Ce^3+^ ions can electrostatically attract negatively charged components of bacterial cell walls, disrupting bacterial cell integrity, interfering with bacterial cell membrane function and metabolic processes, and blocking the bacterial respiratory chain, leading to bacterial death (148, 149). Furthermore, cerium ions participate in the Fenton reaction, promoting the production of hydroxyl radicals, which further damage bacterial membrane structures and enhance the antibacterial effect (150). Cerium-doped bioactive glasses and hydroxyapatite materials not only possess excellent antibacterial properties but also promote the growth and differentiation of bone cells, enhancing the biocompatibility and bone regeneration ability of bone tissue (151, 152). For example, cerium ion-doped hydroxyapatite exhibits significant antibacterial activity against both Gram-positive and Gram-negative bacterial strains while maintaining good osteocyte compatibility (152).

The role of cerium ions in bone immunomodulation is primarily manifested in their ability to regulate macrophage polarization. Research shows that cerium ions promote the differentiation of M2-type macrophages, enhancing their ability to secrete anti-inflammatory cytokines such as IL-10 and TGF-β, thereby optimizing the bone immune microenvironment and promoting tissue reconstruction and repair. Concurrently, cerium ions inhibit the activation of M1-type macrophages by influencing the NF-κB pathway, reducing the release of pro-inflammatory factors such as IL-1β and TNF-α (153, 154), thereby suppressing excessive inflammatory responses and protecting bone tissue from inflammatory damage. By scavenging ROS and RNS, cerium ions significantly reduce the level of oxidative stress in the surrounding inflammatory microenvironment, indirectly affecting macrophage polarization (155). For example, green-synthesized cerium oxide nanoparticles induced M2 phenotype polarization of macrophages *in vitro*, significantly upregulating the expression of IL-10, IL-4, and TGF-β, while also inhibiting the production of major pro-inflammatory mediators like TNF-α and IL-6, promoting wound healing (156). This is beneficial for attenuating local inflammatory responses, maintaining immune balance, and preventing chronic inflammation. Cerium ions may also have regulatory effects on the immune function of bone matrix MSCs and osteoblasts. By regulating the bone immune microenvironment of MSCs, cerium ions promote the secretion of anti-inflammatory cytokines by MSCs, thereby enhancing osteoblast function and bone formation, and improving the self-repair capacity of bone tissue (155).

#### 2.2.7 Zirconium ions

Zirconium ions (Zr^4+^) possess stable physicochemical properties and good biological activity, which has garnered significant attention for their use in bone tissue repair engineering.

Zirconium ions may influence bone metabolism, such as by regulating bone metabolism-related enzymes and signaling pathways, optimizing bone mineralization, and contributing to bone matrix stability and regeneration (157). Studies have found that zirconium-based materials release some zirconium ions after implantation into bone tissue. At the bone matrix, zirconium ions can interact with the mineral and organic components of bone, thereby affecting the biological functions of bone cells and bone tissue metabolism (158, 159). For example, zirconium ions promote the expression of bone formation markers such as bone morphogenetic protein-2 (BMP-2), alkaline phosphatase (ALP), and osteoprotegerin (OPG), facilitating bone repair and regenerative capacity, demonstrating the positive biological activity of zirconium ions in the bone matrix (158). Furthermore, zirconium ions have regulatory effects on bone cells, including osteoblasts, osteoclasts, and BMSCs. The hydrophilicity and surface roughness of zirconium materials indirectly regulate bone cell adhesion, proliferation, and differentiation by controlling the rate of zirconium ion release (157, 159). For instance, the surface of zirconium-containing nanocomposites treated with plasma can significantly enhance the initial adhesion and ALP activity of BMSCs and endothelial cells, promoting bone tissue formation (159).

Regarding the recognition and response of immune cells to zirconium-based material particles, research indicates that zirconium-based nanocoatings not only possess excellent biocompatibility but also can regulate immune cell behavior. For example, zirconium phosphate (ZrP) nanonetwork-modified zirconia surfaces exhibit higher osseointegration ability and also promote macrophage polarization towards the M2 phenotype (160). Zirconium-containing alloys, such as Ta-20Zr alloy, can regulate inflammatory responses, promoting the secretion of IL-6 and IL-10 and helping maintain the balance of M1/M2 macrophages (161), thereby making the local immune environment more conducive to bone tissue growth. Other related studies have shown that active components released from zirconium-based materials, such as fluorine-doped zirconium metal-organic framework (Zr-MOF) films, can regulate gene expression in macrophages, enhancing anti-inflammatory function by inhibiting pro-inflammatory signaling pathways (e.g., NF-κB and IL-6) while upregulating the expression of the anti-inflammatory cytokine IL-4 (162).

Zirconium ions can regulate the bone immune microenvironment by affecting the distribution and function of immune cells. For example, radioactively labeled zirconium isotopes (e.g., Zr-89) have been used to track the migration and homing of B cells and plasma cells in the bone marrow, demonstrating the role of zirconium in the bone marrow localization of immune cells (163). This suggests that zirconium ions not only participate in the metabolic activities of bone cells but may also affect the dynamics of immune cells within the bone marrow. Furthermore, the low toxicity and good biocompatibility of zirconium materials offer unique advantages in the field of bone immunomodulation, maintaining bone tissue immune balance, reducing inflammatory responses, and holding significant promise for bone repair (164).

#### 2.2.8 Other bioactive metal ions (Au, Ag, Al, Fe, Li)

Gold (Au) ions and gold nanoparticles exhibit anti-inflammatory properties by inhibiting NF-κB signaling and reducing pro-inflammatory cytokine production in macrophages, which may indirectly benefit bone healing in inflammatory conditions (165, 166). Silver (Ag) ions are well-known for their potent antibacterial activity; however, at controlled concentrations, they can also modulate macrophage polarization toward M2 phenotype and reduce osteoclastogenesis, though their cytotoxicity requires careful management (167, 168). Aluminum (Al) ions, though historically associated with bone toxicity at high doses, have been shown in low concentrations to stimulate osteoblast proliferation via activation of the Wnt/β-catenin pathway, but their immunomodulatory effects remain controversial and need further clarification (169, 170). Iron (Fe) ions play a dual role: iron overload promotes M1 macrophage polarization and ROS production, exacerbating inflammation and bone resorption (171, 172), whereas iron chelation or restriction can induce M2 polarization and support tissue repair (173). Moreover, iron is essential for osteoblast differentiation and collagen synthesis, making its homeostasis critical for bone health (172). Lithium (Li) ions have gained attention for their ability to activate the Wnt/β-catenin signaling pathway, promoting osteoblast differentiation and bone formation (174). Importantly, lithium exerts profound immunomodulatory effects on T cells: it enhances the generation and suppressive function of Tregs while inhibiting Th17 differentiation, thereby reducing inflammatory bone loss (175). Lithium also promotes the proliferation of CD8^+^ T cells and enhances their cytotoxic activity, which may be beneficial in bone infection or tumor settings (176). These emerging roles of lithium in osteoimmunology warrant further investigation.

In summary, different metal ions play unique and complementary roles in bone immunomodulation, covering macrophage polarization, T cell subset regulation, angiogenesis, antioxidant effects, and direct osteogenesis. To facilitate systematic comparison and quick reference, **Table 1** summarizes the primary osteoimmune effects and key mechanisms of each ion, **Table 2** summarizes the effects of different metal ions on various immune cell subsets.

**Table 1:** Key metal ions and their osteoimmune regulatory mechanisms in bone tissue.

Metal ions	Primary osteoimmune effects & mechanisms
Zinc (Zn^2+^)	1. Promotes macrophage polarization toward the anti-inflammatory M2 phenotype and alleviates inflammation. 2. Facilitates angiogenesis and improves the microenvironment at bone defect sites. 3. Directly promotes the osteogenic differentiation of bone marrow MSCs.
Magnesium (Mg^2+^)	1. Regulates macrophage polarization to maintain osteoimmune homeostasis. 2. Inhibits early excessive inflammatory responses, creating favorable conditions for osteogenesis. 3. Serves as a component of degradable implants, providing continuous immune regulation during the degradation process.
Manganese (Mn^2+^)	1. Activates innate immunity (e.g., dendritic cells) and adaptive immunity. 2. Modulates the balance of T-cell subsets (e.g., promoting Th2) to foster an osteogenic immune environment. 3. Facilitates macrophage polarization toward the M2 phenotype.
Strontium (Sr^2+^)	1. Induces macrophage M2 polarization and improves the inflammatory environment. 2. Directly promotes osteoblastic differentiation and bone formation. 3. Inhibits osteoclast activity to maintain bone metabolic balance.
Copper (Cu^2+^)	1. Promotes angiogenesis and improves the nutritional supply to bone tissues. 2. Induces macrophage M2 polarization and modulates T-cell responses. 3. Endows materials with antibacterial properties, controlling infection while optimizing the immune microenvironment.
Cerium (Ce^3+^/Ce^4+^)	1. Exhibits strong antioxidant effects, scavenging free radicals and reducing oxidative stress. 2. Induces macrophage M2 polarization while inhibiting the activation of the pro-inflammatory M1 phenotype. 3. Possesses antibacterial activity, particularly in acidic environments caused by infection.
Zirconium (Zr^4+^)	1. Promotes the expression of osteogenesis-related markers, benefiting bone regeneration. 2. Regulates immune cell (e.g., macrophage) behavior and promotes M2 polarization. 3. Exhibits excellent biocompatibility, aiding in the maintenance of osteoimmune balance.

**Table 2:** Effects of different metal ions on major immune cell subsets.

Metal ions	Macrophage polarization	T cell subset modulation	DC maturation	Neutrophil function
Zinc (Zn^2+^)	M2 promotion	↑Treg, ↓Th17	↑ (via cGAS-STING)	↑ROS, ↓NETosis
Magnesium (Mg^2+^)	M2 promotion	↑Treg, ↓Th17, ↑CD8^+^ metabolism	↓NLRP3	↑chemotaxis, ↓NF-κB
Manganese (Mn^2+^)	M2 promotion	↑Th2, ↓Th1	↑STING, ↑maturation	(limited data)
Strontium (Sr^2+^)	M2 promotion	(indirect)	(limited)	↓excessive chemotaxis
Copper (Cu^2+^)	M1 (early)/M2 (late)	↑Th1, ↓Th17	↑NLRP3	↑IL-8, GRO-α
Cerium (Ce^3+^/Ce^4+^)	M2 promotion, ↓M1	(indirect)	(limited)	(limited)
Zirconium (Zr^4+^)	M2 promotion	(limited)	(limited)	(limited)

“↑” denotes promotion, increase, or upregulation, while “↓” denotes inhibition, decrease, or downregulation of the corresponding immune cell subset activity or function.

### 2.3 Application of metal ion-doped biomaterials in bone immunomodulation

#### 2.3.1 Design of metal ion-releasing bone substitute materials

In recent years, metal ion-releasing bone substitute materials have gradually become a significant research direction in the field of bone repair. Elements such as Zn^2+^, Mg^2+^, and Sr^2+^ are important components of natural bone tissue. They can synergistically promote bone regeneration and possess antibacterial properties by modifying the local immune microenvironment, thereby enhancing the bioactivity of the materials. For example, Zn^2+^-doped β-tricalcium phosphate/polylactic acid composites achieve stable sustained release of ions through lattice ion substitution or surface coating methods. This avoids initial cytotoxicity and prevents insufficient release in later stages, while also regulating bone immunity, promoting osteogenic differentiation, and inhibiting inflammation (177–179).

Designing multifunctional biomaterials to achieve the synergistic effects of antibacterial activity, immunomodulation, and bone regeneration is a new trend in the development of bone substitute materials. Doping hydroxyapatite with antibacterial ions such as Ag, Pd, and F can reduce the risk of implant infection. Pd^2+^ substitution for calcium ions in hydroxyapatite exhibits an antibacterial rate greater than 99.9% against common pathogenic bacteria (180). Fluorine-doped Zr-MOF films can release both fluorine ions and fumaric acid, achieving dual regulation of antibacterial and anti-inflammatory effects (162). Multi-metal ion co-release systems, such as Cu^2+^/Mg^2+^, utilizing multilayer polyelectrolyte membranes for long-term stable release, possess antibacterial, antioxidant, and immunomodulatory functions and promote osteogenic mineralization and angiogenesis, demonstrating good potential for bone regeneration (181). Such composite materials provide new ideas for the design of bone substitute materials. Using rational ion doping and sustained-release systems, metal ion-based bone substitute materials can effectively regulate the bone immune microenvironment to promote bone regeneration, opening up broad clinical application prospects for bone defect repair (179, 182, 183).

#### 2.3.2 Synergistic effects of multi-metal ion composite materials

Multi-metal ion composite materials leverage the synergistic release of key ions such as Mg^2+^, Cu^2+^, and Zn^2+^ to construct a multi-component microenvironmental regulatory network, offering advantages in immunomodulation and bone regeneration. Among these, Mg^2+^ can regulate the bone immune microenvironment, induce M2-type polarization of macrophages, reduce inflammation, and lay the groundwork for osteogenesis (116). Cu^2+^, with its pro-angiogenic and antibacterial effects, is an important component for improving the immune and vascular microenvironment for bone repair (184). Zn^2+^ achieves both immunomodulation and bone formation regulation by promoting osteoblast proliferation and differentiation and inhibiting the release of inflammatory mediators. The core mechanism of action of composite materials is the regulation of local immune responses, i.e., by inducing a shift of immune cells in the bone defect area towards a pro-healing phenotype, reducing chronic inflammation, enhancing osteoblast activity and differentiation efficiency, and accelerating bone regeneration (116, 185).

With the advancement of 3D printing technology, integrating multi-metal ion composite materials with 3D printing has become a new research direction. 3D printing allows for the precise construction of porous structures and specific morphologies of metal-organic composites, which possess high specific surface area and good drug-loading capacity, enabling sustained release of metal ions. This helps in bone tissue repair by regulating the behavior of immune cells and macrophage polarization (184).

### 2.4 Clinical potential and challenges of metal ions in regulating bone immunity

#### 2.4.1 Clinical application prospects

Metal ion-based materials can directly promote bone tissue regeneration and also improve personalized bone repair strategies by regulating the bone immune microenvironment. Taking magnesium ions as an example, they can regulate the polarization state of macrophages, increase the expression of anti-inflammatory and osteogenic factors, and create an immune microenvironment conducive to the proliferation and osteogenic differentiation of bone marrow mesenchymal stem cells, thereby accelerating bone tissue regeneration (56). Another class of biomimetic periosteal materials containing magnesium and zinc ions regulates macrophage transformation to the M2 type through the sustained release of metal ions, promoting angiogenesis and osteoblast differentiation, and improving bone repair outcomes (10). Functionalized composite materials based on metal-organic frameworks can achieve precise release of metal ions, regulate immune cell activity, and promote anti-inflammatory and regenerative processes in bone tissue, showing application prospects for bone defects and bone and joint diseases (184). The development of metal ion-based nanovaccines and nanomedicines, combined with immunomodulatory mechanisms, provides new ideas for the personalized treatment of diseases such as bone tumors and bone infections (97, 186). Metal ion-based materials have broad clinical application prospects in bone defect repair, bone infection control, and osteoporosis treatment. They lay the foundation for achieving personalized and precise bone repair strategies. Future research will focus on elucidating the molecular mechanisms of metal ions in bone immunomodulation, improving the controlled release properties and biocompatibility of materials, and facilitating their clinical translation and application, opening new avenues for the treatment of bone-related diseases (2, 4, 56, 187, 188).

#### 2.4.2 Safety and biocompatibility issues

While metal ions play a crucial role in bone immunomodulation, issues regarding their safety and biocompatibility cannot be ignored. The concentration and release rate of metal ions can bidirectionally regulate immune responses. Appropriate concentrations can promote immune cell activation and bone tissue repair, but excessively fast release or high concentrations can lead to cytotoxic reactions and excessive inflammatory responses. Therefore, controlling the release of metal ions is of utmost importance.

For long-term implants, the safety of metal ion release requires further evaluation. When implants remain in the body for extended periods, corrosion and wear of metallic materials can occur, leading to the release of metal ions or particles, causing local or systemic adverse reactions. For example, nanoparticles detached from titanium alloy implant surfaces can trigger inflammatory responses in local tissues and can circulate to other organs and deposit, potentially causing systemic toxicity (189). Additionally, some metal ions, such as chromium and cobalt, are highly toxic and sensitizing, increasing the risk of immune rejection and implant failure (190). Researchers are using methods such as surface chemical modification, polymer coating, and metal-organic frameworks to control the release rate and concentration of metal ions, improving the stability and biocompatibility of biomaterials. For instance, MOFs achieve sustained and targeted release of metal ions, enhancing bone tissue regeneration while reducing metal ion toxicity (184, 191).

The morphology and particle size of biomaterials can also affect immune responses. Titanium particles of different sizes released during the process can stimulate immune cells to varying degrees; small particles tend to cause inflammatory reactions, while medium-sized particles exhibit better biocompatibility and lower pro-inflammatory activity (192). Therefore, the design of metal ion release systems must consider various factors, including particle size, surface chemistry, and release rate, to achieve an optimal balance between immunomodulation and safety.

### 2.5 Future research directions

#### 2.5.1 Systematic elucidation of the synergistic regulatory mechanisms of multi-metal ions

The cooperative effects of multiple metal ions during bone immunomodulation are receiving increasing attention. They can collectively regulate the immune microenvironment, angiogenesis, and neurogenesis of bone tissue through complex molecular mechanisms, thereby promoting bone repair and regeneration. A recent study combined a magnesium-copper bimetallic organic framework (MgCu-MOF74) with polyphenols such as gallic acid (GA) to prepare a 3D-printed composite scaffold with good porosity. This scaffold effectively releases magnesium and copper ions, exhibiting excellent antioxidant and pro-angiogenic effects for bone. Magnesium ions primarily induce macrophage polarization towards the M2 type by regulating their polarization, thereby reducing inflammatory responses. Copper ions promote endothelial cells to produce more angiogenic factors, improving blood supply to bone tissue, which is beneficial for new bone growth. The release of the two ions enhances the biological effects of single metal ions, creating a collaborative environment that promotes bone regeneration. Gallic acid also provides antioxidant protection to the scaffold, promoting the morphological maturation of Schwann cells, which is beneficial for neuroregeneration (8). The regeneration of the nervous system is interconnected with immunity and angiogenesis, collectively promoting bone tissue repair. The composite scaffold enhances intercellular interactions and improves communication between cells, optimizing the osteogenic microenvironment from multiple aspects.

#### 2.5.2 In-depth investigation of cell-cell communication in the bone immune microenvironment

There are numerous modes of cell-cell communication within the bone immune microenvironment. The development of single-cell sequencing technology has enabled a deeper understanding of cellular interactions in this environment, revealing dynamic communication networks between immune cells and bone cells.

Exosomes, secreted by various cells including tumor cells, contain multiple bioactive molecules (such as proteins, lipids, and nucleic acids) and play a crucial role in bone immunomodulation. In multiple myeloma, it has been found that exosomes secreted by tumor cells affect the activity of immune cells in the bone marrow microenvironment, contributing to the formation of an immunosuppressive microenvironment that favors tumor cell survival and growth (193, 194). Bone cells, immune cells, and MSCs can all secrete exosomes, which mediate immune regulation and bone regeneration processes within bone tissue (195). These exosomes influence the differentiation, migration, and function of recipient cells by transferring specific non-coding RNAs and proteins, thereby regulating bone immunity.

Cytokines play a regulatory role in information exchange between immune cells and bone cells. Studies have shown that cytokines secreted by immune cells in the bone marrow, such as IL-6, IL-1β, TNF-α, and RANKL, can directly affect bone resorption and formation processes (196, 197). These cytokines not only regulate local bone metabolism but also influence the activation and migration of immune cells, thus establishing a complex cellular communication network.

Using single-cell RNA sequencing and computational analysis tools to investigate cell-cell communication in the bone immune microenvironment, for example, in the bone marrow and bone tumor immune microenvironment, single-cell analysis reveals that CD8^+^ T cells, MDSCs, and macrophages interact with MDSCs, macrophages, and CD8^+^ T cells through specific ligand-receptor systems, affecting their immune activity and bone metabolism (198, 199). Ligand-receptor analysis tools such as CellphoneDB and CellChat are widely used to analyze these cellular communications, identifying many important regulatory molecules, such as JAG1-NOTCH1/2 and CXCLs-CXCRs, and uncovering signaling pathways between immune cells and bone-forming cells (200, 201).

Neuro-immune communication is an important component of the bone immune microenvironment, regulating immune responses through interactions between neurotransmitters and immune cell receptors. The adrenergic receptor signaling pathway regulates inflammatory responses in immune cells, thereby affecting bone immune homeostasis (202). Neurotransmitters indirectly participate in bone repair and immunomodulation processes by influencing the migration and activation of immune cells.

Aberrant cell-cell communication is closely associated with various bone immune-related diseases. In diseases such as leukemia and multiple myeloma, the cellular communication network is reshaped, promoting immune escape and tumor development (203, 204). Interventions targeting these communication pathways, such as blocking the PD-1/TIGIT signaling axis or regulating exosome release, have become new therapeutic strategies (193, 205).

#### 2.5.3 Clinical translation and personalized treatment strategies

In recent years, metal ion-based bone repair materials, owing to their multi-functional regulatory roles in bone tissue regeneration, have gradually become a research hotspot in the field of bone immunomodulation. To facilitate their clinical application, systematic clinical trials are needed to comprehensively evaluate their efficacy and safety. Mg, as a degradable metal implant, can promote osteogenesis and inhibit osteoclast activity, and has been preliminarily applied in clinical orthopedic treatment. However, it is still in the research stage, and its biological evaluation system and phenotypic improvement require further refinement (4). Additionally, antibacterial bone materials for infected bone defects hold great potential for clinical translation. By releasing antibacterial metal ions, surface modification, or combining multiple materials, these materials can effectively control infection and promote bone repair, improving treatment outcomes (188). However, most current research is still at the basic and animal experimental stages, lacking support from large-scale, multi-center clinical data, which limits its application. Promoting clinical trials and randomized controlled trials of metal ion-based bone repair materials is an important pathway to achieving safe and effective translation.

On the other hand, individual differences in bone immunomodulation make personalized treatment strategies particularly important. The immune status of patients with bone injuries or diseases varies greatly. Differences in the dynamic changes of immune cells and the local immune microenvironment directly affect the outcome of bone repair (206). Developing personalized bone immunomodulation strategies based on the patient’s immune status has become a new direction in clinical treatment.

## 3 Conclusion

With the deepening integration of osteoimmunology and bone tissue engineering, metal ions hold significant clinical application prospects. The mechanisms by which metal ions affect bone repair are complex, involving the regulation of macrophage polarization, inflammatory responses, immune signaling pathways, and the promotion of bone cell function and bone regeneration, providing new ideas and methods for the treatment of orthopedic diseases. However, this field still faces many challenges. First, the molecular mechanisms by which metal ions regulate bone immunity have not been fully elucidated. There is a lack of systematic and in-depth analysis of the interactions between different metal ions and their effects on various immune cell subsets. Second, although the functionality of biomaterials is continuously improving, their long-term biosafety and stability in complex clinical environments require further validation to ensure reliable and effective application.

Future work should focus on systematically investigating the synergistic regulatory mechanisms of multi-metal ions, exploring the interactions and dynamic balances between different metal ions to maximize their beneficial effects on bone regeneration while minimizing side effects, and promoting the clinical application of metal ion-regulated bone immunity.

Research on the regulation of the bone immune microenvironment by metal ions has opened new avenues for bone tissue engineering and the treatment of bone diseases. Elucidating molecular mechanisms, improving biomaterial design, and enhancing clinical translation are expected to achieve safer, more effective, and more personalized bone regeneration therapies, advancing the clinical translation and development of bone regeneration treatments that promote bone repair by regulating bone immunity.

## Data Availability

The original contributions presented in the study are included in the article/supplementary material. Further inquiries can be directed to the corresponding authors.

## Glossary

ALP: alkaline phosphatase
BMDC: bone marrow-derived dendritic cell
BMP-2: bone morphogenetic protein-2
BMSC: bone marrow mesenchymal stem cell
CaSR: calcium-sensing receptor
CAT: catalase
CTL: cytotoxic T lymphocyte
DC: dendritic cell
G-CSF: granulocyte colony-stimulating factor
GRO-α: growth-regulated oncogene alpha
HIF-1α: hypoxia-inducible factor-1α
HUVEC: human umbilical vein endothelial cell
ICD: immunogenic cell death
IL: interleukin
MAPK: mitogen-activated protein kinase
MCP-1: monocyte chemoattractant protein-1
MDSC: myeloid-derived suppressor cell
MOF: metal-organic framework
MSC: mesenchymal stem cell
MT: metallothionein
NET: neutrophil extracellular trap
NF-κB: nuclear factor kappa-light-chain-enhancer of activated B cells
NK: natural killer
NOX2: NADPH oxidase 2
OPG: osteoprotegerin
OSM: oncostatin M
PI3K: phosphoinositide 3-kinase
PLGA: poly(lactic-co-glycolic acid)
PMN: polymorphonuclear neutrophil
RANKL: receptor activator of nuclear factor-κB ligand
RNS: reactive nitrogen species
ROS: reactive oxygen species
SOD: superoxide dismutase
STAT: signal transducer and activator of transcription
STING: stimulator of interferon genes
TCR: T cell receptor
TGF-β: transforming growth factor-beta
Th: T helper cell
TNF-α: tumor necrosis factor-alpha
Treg: regulatory T cell
TRPM7: transient receptor potential melastatin 7
VEGF: vascular endothelial growth factor.
